# Review of Prominent Cytokines as Superior Therapeutic Targets for Moderate-to-Severe Atopic Dermatitis

**DOI:** 10.7759/cureus.9901

**Published:** 2020-08-20

**Authors:** Zaira Hassan, Enkhmaa Luvsannyam, Dhara Patel, Swetha Nukala, Suvarna Rekha Puvvada, Pousette Hamid

**Affiliations:** 1 Department of Research, California Institute of Behavioral Neurosciences & Psychology, Fairfield, USA; 2 Department of Research, California Instititute of Behavioral Neurosciences & Psychology, Fairfield, USA

**Keywords:** atopic dermatitis, inflammation, cytokines, interleukins

## Abstract

Cytokines predominate the inflammatory pathways in diseases like rhinitis, asthma, and atopic dermatitis. Corticosteroids and immunosuppressants are presently the mainstays of treatment for patients with moderate-to-severe disease, but often accompany a poor side effect profile. In this review, we attempt to consolidate current data on various interleukins (IL) that participate in the pathogenesis of atopic dermatitis (AD) to further improve therapeutic strategies. For now, dupilumab is the most accepted biologic to be registered for treatment for moderate-to-severe disease. Recently, IL-37, IL-13, IL-26, IL-17 & IL-31/33 axis as well as proteins like thymic stromal lymphopoietin (TSLP) show promising results as future therapeutic targets because of their important role in the pathogenesis of AD. However, further studies are required to clarify the safety and efficacy of these interventions compared to current treatment modalities but it is worthwhile to pursue research into biologics as a more successful treatment option for moderate-to-severe AD.

## Introduction and background

**Figure 1:**
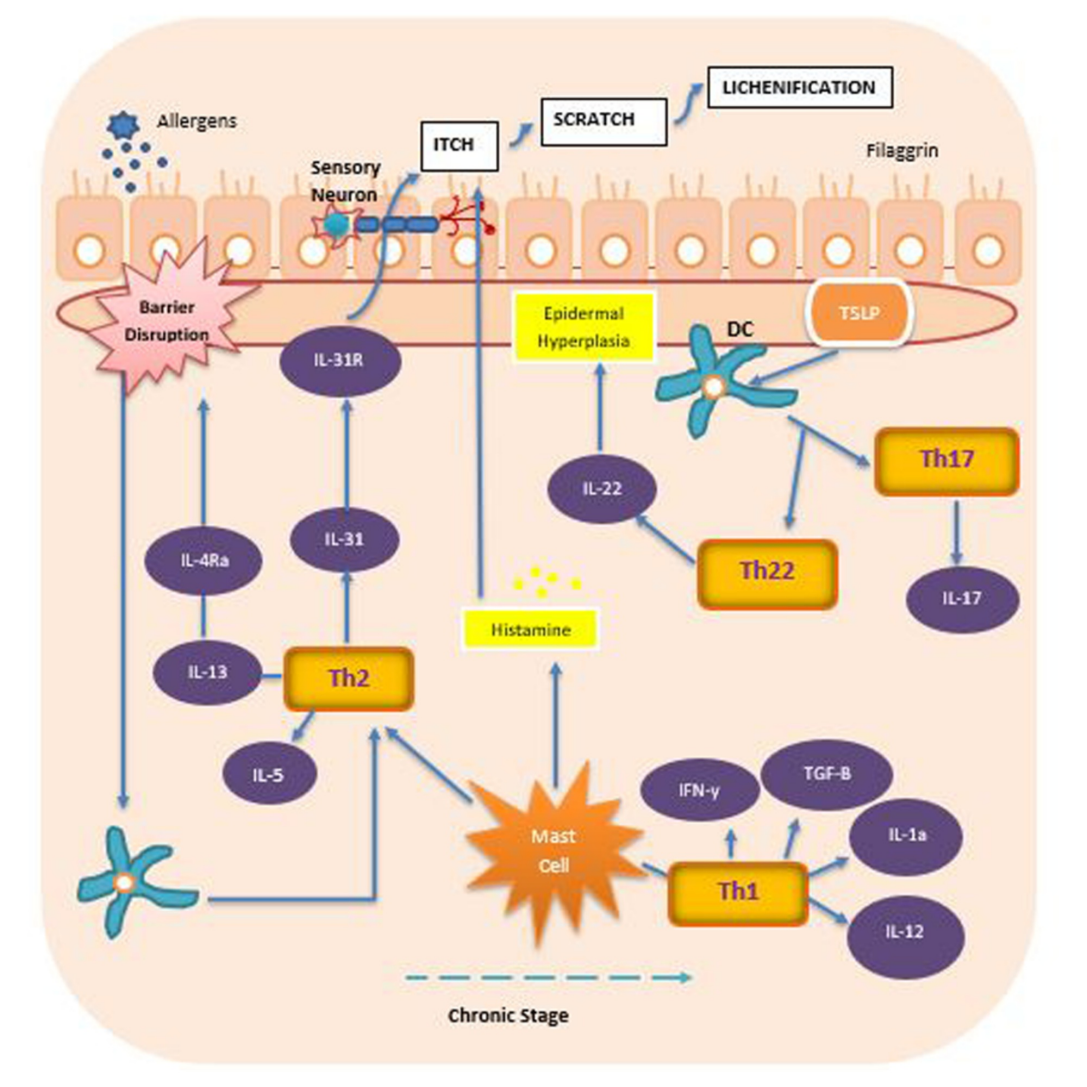
This is a summary of inflammatory cells contributing to the barrier defect, inflammation and pruritis in Atopic Dermatitis Summary of immunological mediators in atopic dermatitis (AD). IL (Interleukin)-4 and IL-13 are produced by Th (T helper)-2 cells [3]. IL-13 downregulates filaggrin, a skin barrier protein, and then induces barrier dysfunction. This dysfunction leads to increased production of TSLP (thymic stromal lymphopoietin) which then stimulates DC (dendritic cells) to affect Th cells to promote further release of inflammatory cytokines [3]. This impaired barrier also allows allergens to enter and promotes water loss from inside. Nerve endings also get activated by immune mediators and cytokines like IL-31 which leads to intense itch-scratch cycle [13]. Histamine, mostly from mast cells, are also a major cause for inducing itch in AD [13]. IL-12 along with IL-18 are involved in Th-1 response to induce the production of IFN-ƴ (interferon gamma) and TGF-B (transcription growth factor beta) [8]. Type 2 predominance is followed by Th1 response when progressing from acute to chronic lesions [9].

Atopic dermatitis (AD) is a recurrent, chronic inflammatory skin disease [1]. It is characterized by an impaired epidermal barrier, severe skin inflammation, cutaneous infections, and pruritus [2]. It presents with dry eczematous lesions, often accompanied by severe pruritis that has a negative effect on the quality of life and psychosocial wellbeing [3]. These lesions become thick plaques with lichenification in chronic AD [4,5]. It has been found to affect up to 20% of children and about 10% of adults [1] with 60% of pediatric patients experiencing the disease before they complete their first year of life [1]. About 20% of those affected in the general population are found to have moderate-to-severe disease [6]. Along with demographic variations, keratinocytes are also influenced by factors such as environmental allergens, scratching of the area, and bacterial superimposition which lead to the release of inflammatory cytokines and other proteins like thymic stromal lymphopoietin (TSLP) [7] as seen in Figure 1.

T-helper (Th) 2 cells predominate the initial phase of AD which involves interleukin (IL)-4 and IL-13, followed by Th1 response [8]. Infantile or early childhood AD is known to be mediated by increased levels of allergen-specific or total immunoglobulin (Ig) E levels. Progression of AD is through epidermal barrier dysfunction and mutations of the Filaggrin (FLG) gene in addition to abnormal IgE levels [3]. Although they share a strong Th2 (IL-4, IL-13, IL-31) lymphocytic activation, some AD phenotypes (extrinsic, Asian and pediatric) express higher levels of Th22 and Th1 [9]. Extrinsic AD also shows increased IgE production [8,10]. Th17 and Th9 cytokines (IL-17, IL-12/IL-23, IL-9) seem to predominate intrinsic AD [8]. IL-37 is a noteworthy cytokine because it is found to inhibit IL-1, IL-6, IL-8, IL-17, and IL-23 which are known pro-inflammatory cytokines [11]. It has been suggested by Lee et al. that IL-18 is known to activate mast cells to release chymase which cleave pro-IL-18 to accelerate the inflammatory process in AD lesions and hence release of cytokines [8].

The multifactorial nature of AD, both environmental and genetic, as well as chronic relapse of the disease has made it difficult to establish an effective treatment strategy and justifies the need to search for novel and optimum solutions. Topical emollients, antihistamines, and glucocorticoids, along with calcineurin inhibitors, are the mainstay of treatment for mild disease but only offer temporary relief [12]. Thirty-three percent of AD patients with moderate-to-severe disease require systemic treatments like oral steroids, methotrexate, ultraviolet type B (UVB) phototherapy and other immunosuppressants [12]. Unfortunately, these agents are not suitable for long term use due to their prominent adverse effects including increased liver enzymes [13]. Although Th2 targets such as dupilumab have shown benefit in AD, many patients still do not achieve disease resolution as shown by Eczema Area and Severity Index-90 (EASI) results approximating 30% [9]. Figure 1 shows a schematic summary of some important aforementioned players in the pathogenesis of AD. 

Therefore, it becomes pertinent to pursue an investigation on developing treatments that focus on other immunological targets like IL-37, IL-13, IL-26, IL-17 & IL-31/33 axis as well as proteins like thymic stromal lymphopoietin (TSLP). Such immunological factors have been gaining more importance over the past few years due to their critical roles in the initiation as well as progression of AD. In this article we attempt to consolidate data on various cytokines as well as introduce other molecular players that promote inflammation in AD in the hopes to achieve more targeted immunological benefits for patients suffering with moderate-to-severe disease. These biologics may evidently prove to be superior in efficacy while also maintain a tolerable side effect profile as compared to current immunosuppressants.

## Review

Method

A search was conducted using PubMed and Google Scholar database of articles with restrictions of English language, free articles, articles published in the last 10 years (2010-2020), and we included both human and animal studies. Abstracts and full-text articles containing the keywords “atopic dermatitis AND inflammation; atopic dermatitis AND interleukins AND inflammatory response; eczema AND treatment AND cytokines." Following this, of the 261 articles, 42 were used for the final review.

Results

Various cytokines were analyzed that are presently serving as a mode of treatment or currently being studied to become potential treatments, as seen in Table 1. Table 2 depicts other cytokines which have immense potential in being considered as a biologic agent in the future because of their deep impact in the inflammatory pathway.

**Table 1:** Current biologics under consideration as treatment for moderate-to-severe atopic dermatitis HTN, Hypertension; IL, Interleukin; TRPA1, Transient Receptor Potential Ankyrin 1; AE, Adverse Event; URI, Upper Respiratory Infection; Th, T-helper; AD, Atopic Dermatitis; CPK, Creatine Phosphokinase; IFN-y, Interferon gamma; SCORAD, Severity Scoring of Atopic Dermatitis Index; TSLP, Thymic Stromal Lymphopoietin

Interleukin or Protein	Mechanism of Inflammation	Established or prospective treatment	Safety
IL-2	T cell activation through autocrine pathway, cause of itch [13]	Cyclosporine – inhibition of calcineurin activation [13]	Recommended use should not exceed 1-2 years. Side effects: nephrotoxicity, HTN, tremors, Rare: Lymphoma [14]
IL-13	Activation of TRPA1 [12]	Lebrikizumab – Phase II trial, topical cream [12]	No life threatening AEs [12]
		Tralokinumab – Phase II treatment was associated with promising results [12]. Phase III has met endpoints [15]	Most common AEs headache and URI. Acceptable tolerability profile [12]
IL-4	Th2-mediated immune response [12]	Dupilumab – Phase III, On the market as Dupixent [12]	Increased rate in allergic conjunctivitis [12]
IL-31	Binds to IL-31 receptor [IL-31R] complex, involved in mediation of inflammatory itch [16]	Nemolizumab – Phase II B, for pruritus associated with AD [17]	Elevations in CPK and asthma exacerbations noted [17]
IL-22	Part of the IL-10 family, produced by Th22 cells [18]. Levels increased in inflamed skin from patients with psoriasis, AD, and allergic contact dermatitis [19]	ILV-094 – Phase II, ongoing [13]	Not available
IL-12/23	Induces Th1 response, resulting in production of IFN-y [8]	Ustekinumab – Phase II Study [20]	Superior response in treatment group vs control group – SCORAD50 = 31% and 16 % respectively [20]
IL-17	Secreted by Th17. IL-17A-IL-17F cytokines possess proper receptors. Involved in both Psoriasis and AD [21]	Secukinumab – targets IL-17A Phase II ongoing [13]	Not available
		MOR106 – targets IL-17C Phase I [13]	
TSLP	Similar structure to IL-17, Induces Th2 inflammation	AMG157 – Phase I trial [15] Tezepelumab – Phase II [22]	Results not published [22]
		MK8226 – Phase I trial against TSLP receptor [13]	

**Table 2:** Cytokines to consider as future therapeutic targets IL, Interleukin; JAK STAT, Janus Kinase Signal Transducer and activator of transcription; CCL2, Chemokine Ligand 2; AD, Atopic Dermatitis; EASI, Eczema Area and Severity Index

Interleukin	Mechanism of action
IL-24	Belongs to IL-20 family, with Type I and type II receptors. Both are expressed on non-immune cells like keratinocytes, bronchial epithelial cells [23]. Activates the JAK STAT pathway. Signaling suppresses dermal production of CCL2 and reduces inflammation [23]
IL-18	Stimulates both Th2 and Th1 response. Released by keratinocytes and inflammatory dendritic cell. Elevated expression is involved in pathogenesis of AD in adults, children and mouse models. Can also enhance production of IL-4, IL-5, IL-9, and IL-13 [8]
IL-37	Belongs to IL-1 family. Binds to IL-18R, recruits IL-1R8 to form IL-37/IL1R8/IL-18Rα complex. Complex promotes anti-inflammatory signaling pathways [11].
IL-19	Stimulates the production of Th2. Belongs to the IL-10 family of cytokines. Under the influence of IL-17A, IL-19 is strongly expressed in AD lesional skin [21].
IL-26	IL-20 family, signals through STAT1 and STAT3, strongly expressed by Th17 cells [1]. Higher in pediatric vs adult AD [1]
IL-33	Belongs to IL-1 family, promotes Th2 inflammation. Expression is increased in keratinocytes of AD lesions. Serum IL-33 level highly associated with EASI score. IL-33 levels reduced after clinical improvement of skin lesions [24]

Discussion

IL-31/IL-33 axis

Both IL-31 and IL-33 are members of the alarmins family. Cytokines in this family contain several endogenous peptides and proteins that are involved in cellular damage, apoptosis along with adaptive and long term immune memory [25]. IL-33 functions both as a nuclear factor to regulate gene expression intracellularly and regulates gene expression as an IL-1 family cytokine extracellularly [26]. With its IL-1 domain, IL-33 is able to form an IL-33/ST2/IL1RAcP complex which induces the intracellular signaling of various kinases and tumor necrosis factors, thus promoting the inflammatory cascade [27]. It was discovered by Di Salvo et al. that the two cytokines played an important role in both allergic and inflammatory diseases [26]. Also, that a relationship exists between them in that the induction of one cytokine will stimulate the induction of another. In a study by Tamaqawa et al., IL-33 was higher in the serum of patients with AD than the serum of those who were healthy, had psoriasis or urticaria. IL-33 levels also correlated with disease severity [24]. The activation of the IL-33/ST2 complex involving Th2/IL-31 immune response also plays a key role in the development of allergic inflammation, such as that seen in asthma [24]. It has been postulated that the dosage of IL-33 can be crucial in therapeutic efficacy, not only in atopic dermatitis but also in other autoimmune diseases as well [16]. Nemolizumab is a monoclonal antibody that binds the IL-31 receptor alpha and blocks IL-31 from affecting neurons. This inadvertently reduces the sensation of pruritis [17]. Nemolizumab may not improve eczematous lesion as much as dupilumab, but IL‐31 seemed to improve pruritus more efficiently and rapidly. Therefore, blocking IL-31 may prove to be efficacious in the itch-scratch cycle of AD [28].

IL-4 & IL-13

IL-4 and IL-13 gene expression is greater upregulated in pediatric and adult AD skin lesions compared with those with healthy skin [3]. These cytokines stimulate B cells to produce IgE, are involved in the epidermal barrier dysfunction, promote type 2 lymphocyte driven inflammation, ultimately leading to atopic disease [3]. It has been found that higher levels of IL-13 producing cells in the cutaneous lymphocyte antigen (CLA)+ T helper cell population are present for both pediatric and adult AD patients than in healthy controls [29]. Both IL-4 and IL-13 have recently been shown to play prominent roles due to the superb treatment response to the anti‐IL‐4 receptor α (IL‐4Rα, IL4R) antibody dupilumab, which inhibits both IL-13 and IL-4 [30]. Tralokinumab, anti-IL-13 antibody, has also shown promising results in successfully improving eczematous lesions [27]. Dupilumab is available on the market under the brand name Dupixent and is used to treat moderate-to-severe AD in patients over six years of age [30]. As of December 2019, tralokinumab has met secondary endpoints as a phase III trial but not received authorizations by regulatory agencies at this point [15]. Barrier dysfunction and skin inflammation in AD are also affected by periostin, a nonstructural protein present in the extracellular matrix, induced by IL‐4 or IL‐13 [31,32]. Periostin is known to increase production of Thymic Stromal Lymphopoetin (TSLP) and stimulate proliferation of keratinocytes. In a mite-induced AD model, it was discovered that the absence of periostin reduced epidermal hyperplasia [33]. The increased expression of periostin in areas of inflammation and injury can thus also prove to be a therapeutic target for drug development.

IL-13 & OVOL1

To maintain skin barrier integrity, expression of FLG and other epidermal proteins is known to play a key role [34]. Ovo-like 1 (OVOL1) is an important player in regulating FLG expression [35]. In a meta-analysis of genomic studies, among the 31 gene loci, the genes of FLG, OVOL1 and IL-13 were found to be substantially associated with AD [36]. Inactivation of OVOL1 downregulates induction of FLG by IL-4/IL-13 [37]. On the other hand, OVOL1 activation upregulates FLG expression [35]. In the study by Tsuji et al., it was noted that IL-4 and IL-13 were involved in inhibiting FLG expression by interrupting OVOL1 signaling [35]. When skin barrier integrity is disrupted, epidermal keratinocytes produce increasing amounts of TSLP, IL-25, and IL-33 which also promotes Th2 cells to produce IL-13 [38]. Hence ensues a fierce cycle that promotes atopic dry skin. This is favorable to the role of IL-13-OVOL1-FLG axis towards the progression of AD.

IL-17 & Thymic Stromal Lymphopoeitin

IL-17 has been known to be a proinflammatory cytokine, mostly involved in inflammatory diseases like psoriasis, arthritis, and inflammatory bowel disease but in recent years has also been known to predominate in patients with intrinsic AD [21]. IL-17 is produced by cluster of differentiation (CD) 4+ Th-17 cells and regulates keratinocyte expression of chemokines [18]. It maintains a cycle in the epithelium in order to strengthen immune barriers [39]. In a study by Koga et al., it was discovered that the peripheral blood and skin lesions of AD patients is affected by IL-17 in the production of cytokines and vascular endothelial growth factor (VEGF). Th17 cells (produces IL-17) were also found to be elevated in these patients and found to be a possible enhancer of the lesions in AD [21]. Secukinumab, a monoclonal antibody against IL-17A, was originally registered for treatment of psoriasis, ankylosing spondylitis, and psoriatic arthritis. In recent light of intrinsic AD characterized by Th17 and Th9 lymphocytes, the subgroup of AD patients with increased expression of Th17 can also benefit from this biologic. A randomized, double-blind trial was conducted as a Phase II trial for the evaluation of secukinumab for AD patients and further trials need to be conducted to assess its efficacy for AD [21]. It has also been observed that the neutralization of IL-17C led to reductions in IL-4 levels, mast cells, and IgE level [40]. This leads to the notion that IL-17C contributes to the atopic inflammatory process in both the initiation and maintenance of atopic disease. MOR106, antibodies against IL-17C was evaluated in a Phase I trial where 83% of patients achieved EASI-50 by the fourth week [21]. TSLP, an epidermal protein, is structured similarly to IL-17 and has been found to induce Th2 inflammatory responses and is elevated in patients with AD [13]. It has been hypothesized that the production of TSLP in the keratinocytes of AD lesions may play a key role in the pathway that leads to asthma from AD [13].

IL-37

IL-37 is considered a natural suppressor of inherent processes of inflammation and immune-mediated responses with both intracellular and extracellular properties [41]. Along with other inflammatory diseases, IL-37 has also found to be elevated in AD patients and also correlates with disease severity [41]. The dual functionality of IL-37 helps to inhibit cytokines that promote inflammation (IL-1α, IL-1 β, IL-1Ra, IL-6, IL-8, IL-17, IL-23, TNF-α, and IFN-γ) and stimulates expression of transcription growth factor (TGF)-β1 and nitric oxide [11]. It also exerts anti-inflammatory effects by regulating and shifting kinases toward anti-inflammatory pathways [11]. Some evidence also links IL-37 to interacting with autophagy. It has been established that AD is often associated with other atopic disorders like asthma, allergic rhinitis, and cardiovascular diseases which indicates similarities in genetic factors [42]. IL-37 has the potential to block allergic inflammation in asthma by recruiting eosinophils and neutrophils and suppressing inflammatory mediators in the Th1/Th2/Th17 pathway. Biologics currently on the market target IL-4 and IL-13 for moderate to severe AD. It can be hypothesized that IL-37 can be used as a biologic to treat AD through inhibition of IL-4 and IL-13 [11]. IL-37 also is implicated in the inflammatory process of other disease processes like psoriasis, psoriatic arthritis, atherosclerotic process like lipid metabolism and apoptosis [11]. This suggests that IL-37 is a potential target of therapy for various diseases due to its great subjugation of inflammatory processes and regulation in immunity. However, further research is still required to understand the expression of IL-37 in the circulation and skin lesions of patients of AD.

Limitations

The review presented here has potential limitations. The review is by no means an exhaustive list of the cytokines and proteins involved in mediating inflammation in AD due to the filters applied in the search engines and only using data from abstracts and free articles. Further addition of references and sources may have added valuable information to expands upon cytokines presented in this article as well as discuss other cells and players that may prove to be superior targets than the cytokines discussed. With additional references, more emerging trials and interventions being studied could have been compared for safety and efficacy. Another essential limitation is that animal studies, specifically data from mouse models were used to prove the involvement of cytokines in AD. Even though animal research provides a rationale for conducting human trials, responses seen in animal studies may not translate into human research and thus may not be used to extrapolate human response.

Future Considerations

Enormous benefit exists in pursuing these cytokines as therapeutic agents. More organized studies will undoubtedly uncover the valuable potential of these cytokines in the pathogenesis of AD. The additional knowledge and treatment modalities will also prove to be advantageous for treating other diseases like psoriasis and rheumatoid arthritis due to cytokines like IL-37 and IL-17 also being heavily involved in this inflammatory process as well [9,11]. Analyzing cytokines may also provide more insight into how each antibody developed behaves differently for each subtype of AD.

## Conclusions

The effects, demographics and pathogenesis of AD is complex and thus emphasizes the need to individualize treatment specially for those with moderate-to-severe disease. Patients with AD suffer from barrier dysfunction and are susceptible to environmental triggers and pathogens which leads to a poor quality of life. Inflammation is predominantly mediated by Th2/Th22 whereas Th1 and Th17 mediate the progression of AD. After much deliberation and efforts by the scientific community, dupilumab, a biologic against IL-4, was registered as a treatment in the United States. More recent data show promising results when it comes to IL-31/IL-33, IL-13, IL-17, IL-37, and TSLP. IL-26 seems more promising for the pediatric population. Larger scale human-based studies still need to be conducted to prove the safety and efficacy of these cytokines as treatments for moderate-to-severe AD.
